# Over-expression of TLR4-CD14, pro-inflammatory cytokines, metabolic markers and NEFAs in obese non-diabetic Mexicans

**DOI:** 10.1186/s12950-014-0039-y

**Published:** 2014-11-29

**Authors:** Cesar Octavio De Loera-Rodriguez, Vidal Delgado-Rizo, Anabell Alvarado-Navarro, Juan Manuel Agraz-Cibrian, Jorge E Segura-Ortega, Mary Fafutis-Morris

**Affiliations:** Doctorado en Ciencias Biomédicas, Guadalajara, Jal, México; Departamento de Fisiología, Centro Universitario de Ciencias de la Salud, Universidad de Guadalajara, Guadalajara, Jal, México; Servicio de Gastroenterología, Antiguo Hospital Civil de Guadalajara ¨Fray Antonio Alcalde¨, Guadalajara, Jal, México

**Keywords:** TLR4, NEFAs, Inflammatory cytokines, Obesity, Metabolic markers

## Abstract

**Introduction:**

Obesity is the world’s most important public health problem. Adipose tissue contributes significantly to increase pro-inflammatory mediators whose cascade begins with the union of TLR4 to its microbial ligands (TLR: Toll Like Receptors). It has been reported recently that NEFAs (Non-Esterified Fatty Acids) bind to this receptor as agonists. The purpose of our study was to determine the levels of expression of TLR4-CD14, the pro-inflammatory cytokines, the metabolic markers and the NEFAs in a group of adult, non-diabetic obese Mexicans.

**Method:**

A group of 210 adult middle-class Mexican non-diabetic obese patients was evaluated: 105 normal weight individuals, and 105 non-diabetic obese. On both groups, the following was tested in each patient: TLR4-CD14 receptors on monocytes in peripheral blood, inflammatory profile, HOMA-IR (Homeostasis Model Assessment-Insulin Resistance), NEFAs and each individual’s anthropometric profile.

**Results:**

Obesity is strongly associated with the expression of TLR4 (77%, MFI (Mean Fluorescence Index) 7.70) and CD14 (86% MFI 1.61) with 66% double positives (*p* = 0.000). These figures contrast with those for the normal weight individuals that constituted the control group: TLR4 (70% MFI 6.41) and CD14 (84% MFI 1.25) with 59% double positives. As for cytokine concentration, non- diabetic obese individuals *vs* the normal weight/thin, the numbers were: IL-1β = 2.0 *vs* 2.5 pg/ml (*p* = NS), IL-6 = 36 *vs* 28 pg/ml (*p* = 0.030), IL-8 = 27 *vs* 27 pg/ml (*p* = NS), IL-10 = 8.4 *vs* 6.8 pg/ml (*p* = NS), TNF-α =31 *vs* 15 pg/ml (*p* = 0.000) respectively. Insulin levels were 12.1 *vs* 19.7 mcU/ml (*p* = 0.000) and the NEFAs were much higher in the obese *vs* normal weight/thin individuals (*p* = 0.000).

**Conclusion:**

Adipose tissue used to be thought of as mere storage of fats and energy, but it has been revealed to be an important neuro-immune-endocrine organ. Immune cells, stimulated by NEFAs, produce pro-inflammatory cytokines, which have a direct effect on oxidating radicals that directly target the release of noradrenalin. This in turn, reactivates the vicious cycle of low-grade chronic inflammation, as is now described in obesity.

## Background

Obesity is a world-wide problem that increases rapidly and can now be considered pandemic. It is considered the main public health problem facing Mexico (ENSANUT 2012). Obese patients have been observed to suffer from chronic low-grade inflammation as a result of the increase in the mass of adipose tissue, which raises the production of pro-inflammatory mediators under exogenous and/or endogenous stimulation [1-3].

Adipose tissue (AT) used to be considered only as reserve tissue for fat. It is now considered an endocrine organ capable of releasing hormones (adiponectin, leptin, resistin, cytokines, chemokines, etc.). Additionally, AT expresses specific receptors for these hormones and for other molecules. In persons of normal weight, the AT is structured with normal size adipocytes, with very little macrophage infiltration. Obese individuals, however, present with hypertrophied adipocytes and elevation of the number of macrophages, both of which contribute to low-grade systemic inflammation [4-6]. It is assumed that adipocyte hypertrophy and local hypoxia are two of the most important factors that contribute to the accumulation of macrophages in the AT of the obese [7,8]. The macrophages of the adipose tissue (ATM) of the obese subjects are often found arranged in “crown-like structure” (CLS) surrounding dead adipocytes [9].

The phenotype of macrophages divides into subsets pro-(M1) or anti-inflammatory (M2). M1 phenotypes or classically activated macrophages are induced by (lipopolyssacaride) LPS and TNF-α and they produce pro-inflammatory cytokines (IL-1, IL-6 y TNF- α). IL-6 is a cytokine relased by macrophages stimulated by noradrenalin (NA) as part of the hypothalamus-hypophysis-adrenal axis. This in turn releases corticosterone, modulating the systemic inflammatory response. However, it has recently been demonstrated in obese rats that the levels of NA are increased and the feedback mechanism NA-IL-6 is disrupted, maintaining the levels of IL-6 even in the presence of corticosterone [10]. Thermic shock protein Hsp-72 is also a stimuli for macrophages to release inflammatory cytokines from both normal weight and obese rats [11].

Phenotype M2 or alternately activated macrophages are induced by glucocorticoids, lL-4 and IL-10 and they produce anti-inflammatory cytokines [8,12-14]. An intermediate phenotype has also been described that is similar to the anti-inflammatory M2 but also secretes great quantities of pro-inflammatory cytokines [15,16]. In obese rats, the increase in numbers of macrophages in the AT is similar to that which occurs in humans. In models of rodents with diet induced obesity, there is a change in the ATM phenotype from an M2-polarized state in lean tissue, to a polarized-M1 state in obese animals [17,18].

In obesity, adipocytes can release pro-inflammatory mediators, such as quimiokine CC (CCL) -2, TNF-α and NEFAs instead of leptin and adiponectine that promote insulin sensitivity in a normal state [19]. Adipose tissue, then, significantly contributes to systemic inflammatory processes [20,21] and to insulin resistance and hyperinsulinemia. We therefore find a highly coordinated.

Association between inflammatory pathways and metabolism [22]. This suggests an association between macrophages and free fatty acids [23].

Toll receptors (TLRs) play a critical role in the induction of the innate immune response in mammals via recognition of molecular patterns associated to microbial pathogens [24-27]. We have so far cloned 10 TLRs in humans [28-33]. TLR ligands include lipopolysaccharide (LPS) for TLR4. TLR4 can be activated by non-bacterial agonists such as Hsp60, fibronectin, Taxol, cover proteins of syncytial respiratory virus and by NEFAs [34-39]. TLRs are type 1 transmembrane receptors characterized by the presence of extracellular repeating motifs rich in leucine and a cytoplasmic tail with a homologous domain to the receptor of IL-1 (TIR) that is required to activate the downstream signaling pathways that lead to the activation of nuclear NF-кB that in turn promotes the increase in pro-inflammatory cytokine production [40].

### Objective

The purpose of this study was to determine the expression of TLR4-CD14 in peripheral mononuclear cells, pro-inflammatory cytokines, metabolic markers and NEFAs in the serum of obese non-diabetic mestizo Mexican adults.

## Materials and methods

### Population characteristics

This is an analytical, descriptive study that included 210 middle-class mestizo Mexican residents of Western Mexico. Of these, 105 were normal weigh individuals, 55 men and 50 women. The second group was also 105, 35 men and 70 women, made up of obese individuals that did not present acute, chronic or inflammatory illnesses, nor were they taking any kind of medication. They had all maintained stable weight for at least 6 months. One of the two groups was categorized as obese, with a Body Mass Index (BMI) ≥30 kg/m^2^; the other group had a BMI ≤24.99 kg/m^2^ and was the control group, being of normal weight. The subjects in the obese group were non-diabetic and presented with normal levels of cholesterol. Table 1 shows the clinical, metabolic, and anthropometric characteristics of all the participants in the study.

**Table 1:** **Clinical metabolic and andtropometric characteristic of study subjects**

	**Non diabetic obese N** **=** **105**	**Normal weight N** **=** **105**	**t student**
Gender (male/ female)	35/70	55/50	-
Age (years)	38.5 ± 12.8	33.4 ± 13.4	0.006
Weight (kg)	93.4 ± 17.2	65.8 ± 9.1	0.000
BMI (kg/m2)	34.7 ± .4	23.3 ± 1.5	0.000
Body fat mass (%)	37.3 ± 6.5	23.4 ± 4.0	0.000
WHI	1.1 ± 0.3	0.8 ± 0.9	0.000
AC (cm)	109.2 ± 13.9	83.4 ± 9.6	0.000
HC (cm)	116.3 ± 14.3	98.2 ± 8.2	0.000
Time whit their weight (years)	6.9 ± 5.4	6.4 ± 4.5	0.488
Glucose (mg/dl)	107.2 ± 9.70	81.1 ± 9.1	0.000
Chlosterol (mg/dl)	173.5 ± 40.8	161.7 ± 35.3	0.027
Triglycerides (mg/dl)	168.5 ± 139.5	126.0 ± 76.0	0.007
LDL (mg/dl)	100.2 ± 36.1	93.6 ± 29.9	0.149
HDL (mg/dl)	40.6 ± 11.5	43.1 ± 10.3	0.108
Insulin (mcU/ml)	19.7 ± 13.2	12.1 ± 3.4	0.000
HOMA-IR	5.3 ± 3.7	2.4 ± 0.7	0.000
NEFA (mEq/L)	4.7 ± 4.7	0.3 ± 0.21	0.000

Data are presented as mean ± standard deviatior. *BMI*: Body mass index; *WHI*: Waist-hip index; *AC*: Abdominal circumference; *HC*: Hip circumference; *LDL*: Low density lipoprotein; *HDL*: High density lipoprotein; *NEFA*: Non-esterified fatty acids.

All participants were volunteers and signed an informed consent form. The study was carried out in accordance with the Helsinki Declaration guidelines and with the official Mexican norms in place regarding health issues.

### Data and sample collection

Anthropometric measurements were taken for each participant along with weight and obesity status. The Body Mass Index (BMI) was calculated using weight and body size (kg/m^2^).

### Anthropometric measurements

All anthropometric measurements were taken by the same researcher to insure accuracy. Body Mass and size were measured using a commercial stadiometer at the Nuevo Leon Clinic #220, in Mexico, with a scale close to 0.2 kg and 1.0 cm, respectively. Skin fold measurements were made with a skin fold caliper (Slim Guide Calliper) according to the guidelines of the International Society for the Advancement of Kineanthropometry (ISAK). Waist/hip circumference was taken with a non elastic measuring tape in centimeters (KaWe CE, Kirchner und Welhelm, Germany), according to ISAK guidelines [41].

### Body mass measurement

Body fat percentage was estimated using an anthropometric equation for the general population [42]. For the general population = 0.465 + 0.180 * (S7SF) - 0.0002406 * (S7SF)^2^ + 0.0661 * (age), where S7SF is the sum of the skin folds of the chest, axila, tríceps, subscapular región, abdomen, suprailiac, and mid-thigh, in millimeters, and age is expressed in years. To determine body density, we used the Wilmore *et al*. equation, which for people active in sports is 1.0988 – 0.0005 * (S7SF), where S7SF is the sum of the skin folds from the chest, axilla (armpit), triceps, subscapular region, abdomen, suprailiac area, and caudal mid-thigh in millimeters [41]. Body fat was calculated using the Siri equation (Siri/body density) – 450 [43].

### Measurement of CD14 y TLR-4

Blood samples were obtained after the signing of the informed consent form and were collected in test tubes with heparin to separate the mononuclear cells of peripheral blood (PBMC), and dry tubes to collect serum. The serum samples were stored at −80°C until their use.

CD14 and TLR4 was measured with flow cytometry using anti-CD14 antibodies (eBioscience, San Diego, CA, USA) and anti TLR4-PE (eBioscience, San Diego, Ca, USA). Appropriate isotope controls were used for each fluorochrome with irrelevant antibodies.

**Figure 1:**
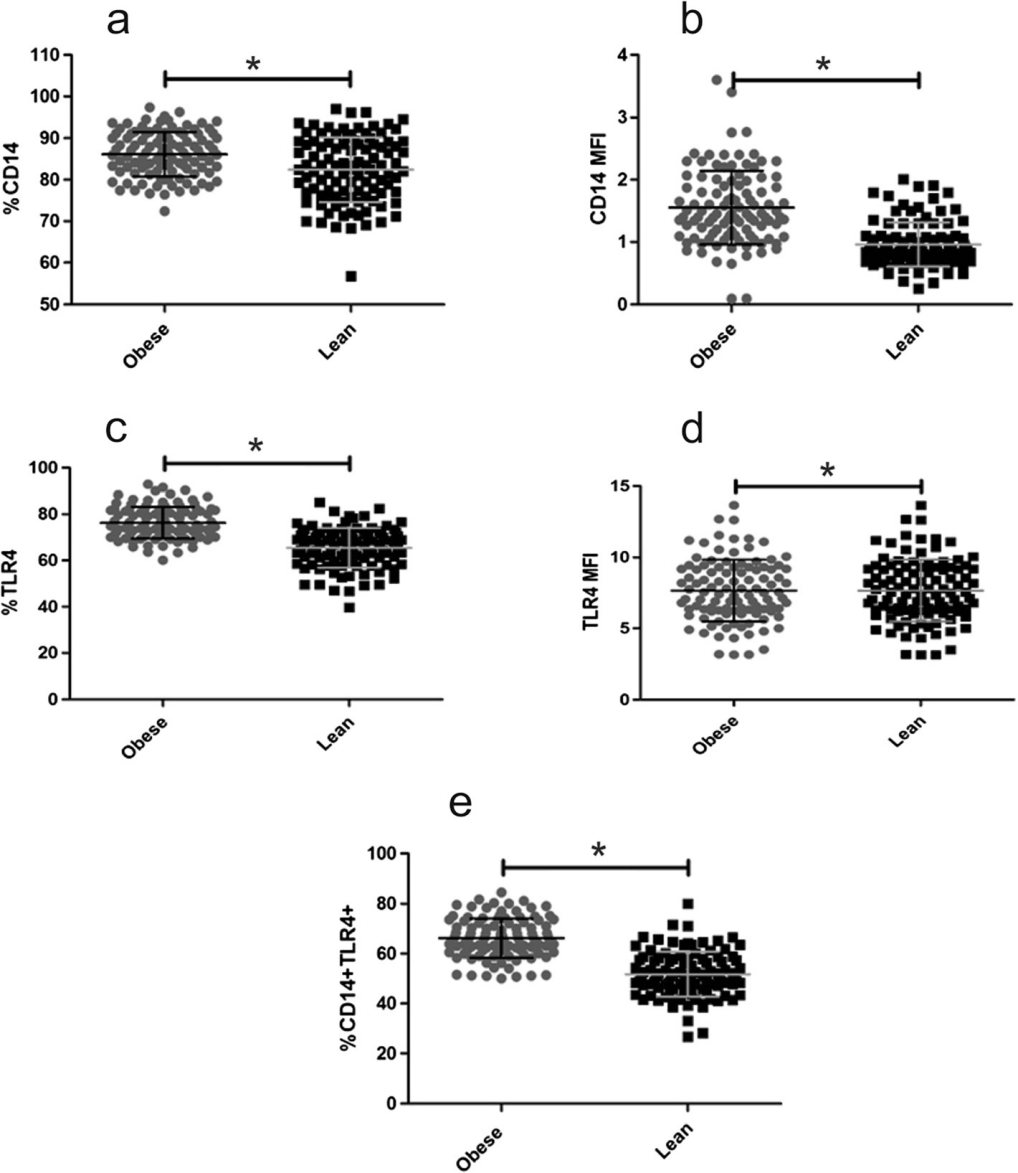
**Percentage and IFM of expression TLR4 and CD14.** The expression of TLR4/CD14 was more in obese then lean individuals. **a)** Percentage CD14 expression. **b)** CD 14 mean fluorescence index. **c)** Percentage TLR4 expression. **d)** TLR4 mean fluorescence index. **e)** Percentage of positive double expression CD14-TLR4. The graphs show the mean ± SD of the results.

Monocyte population was identified based on size and granular characteristics in an EPICS XL-MCL flow cytometer (Beckman Coulter, Krefeld, Germany). A total of 10,000 CD14 + monocytes was read for each sample. The percentage of TLR4 + CD14+ monocytes was calculated from the analysis of the two fluorochromes (See Figure 1). Data was analyzed using FlowJo software (Arbol Star Inc., Ashland, Oregon, USA). Mean Fluorescence intensity (MFI) was determined subtracting the intensity of the geometric mean fluorescence from the sample control isotope.

### Metabolic parameters

All subjects underwent the same 12 hour fasting conditions before testing. Total NEFA levels in serum were measured by enzymatic assay (Wako, Richmond, VA). Insulin levels were measured using an ELISA kit (Genway Biotech, Inc), and glucose levels were measured using a glucometer (active Accu-Chek, Roche).

### Cytokines

Pro-inflammatory cytokines (IL-1β, IL-6, IL-8 and TNFα) and the anti-inflammatory profile (IL-10) were measured using ELISA MAX™ Deluxe Sets kits (BioLegend Inc, San Diego, CA), according to manufacturer’s instructions.

### Statistics

Statistical analysis was made using the SPSS v18.0 program and Excel 2010 software. Tests for significant differences were made, comparing obese to normal weight. Simple statistical comparisons were made using Student’s “t” test. Differences were considered significant when P <0,05 (*). All results are presented as +/− standard deviation SD from the mean.

## Results

### Obesity and clinical and anthropometric characteristics

The clinical and anthropometric characteristics were significantly greater in the obese tan in the normal weight subjects (p <0,001) (Table 1). Mean BMI in the obese group was 34.7+/− 5.4 kg/m^2^ and in the normal weight group, 23,3+/− 1,5 kg/m^2^ (p <0,000); percentage of fat mass was 37,3 +/− 6,5% *vs* 23,4+/− 4% (p <0,000); waist circumference 109,2+/− 13,9 cm *vs* 83.4 +/−9.6 cm (p <0,000); glucose 107,2+/− 9,7 mg/dl *vs* 81,08+/− 9,1 mg/dl (p <0,000); triglycerides 168.5 +/−139.5 mg/dL *vs* 126 +/−76 mg/dL (p <0,000); insulin 19,7+/− 13,2 MCU/ml *vs* 12,1 +/−3,4 MCU/ml (p <0,007) and NEFA 4,7 +/−4,7 mEq/L *vs* 0,25+/− 0,21 mEq/L (p <0,000), respectively.

### TLR4-CD14 expression

The percentage of TLR4 and CD14 expression in monocytes of peripheral blood and the Mean Fluorescence Index (MFI) was significantly greater in the obese (p <0,05) than in the subjects of normal weight (Figure 1).

### Inflammatory profile

**Figure 2:**
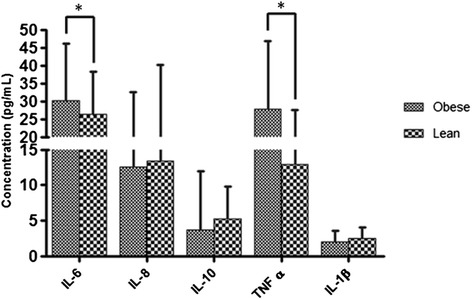
**Serum cytokine concentration.** The serum cytokine from lean and obese groups were measured by ELISA. Cytokines expressions are shown in pg/mL for each group. Results expressed the cytokines levels on serum and are represented as means ± SD values. A strong association of IL-6 and TNF-α with obesity was observed (*p < 0.05).

The concentration of IL-6 and TNFα (36 pg/ml *vs* 31 pg/ml) in serum was greater in the obese and was statistically more significant in comparison to the individuals of normal weight (28 pg/ml *vs* 15 pg/ml), respectively (p <0,05). The concentration of IL-1 β in serum, IL-8, and IL-10 were not statistically significant when comparing obese and non- obese individuals (Figure 2).

### Metabolic markers

**Figure 3:**
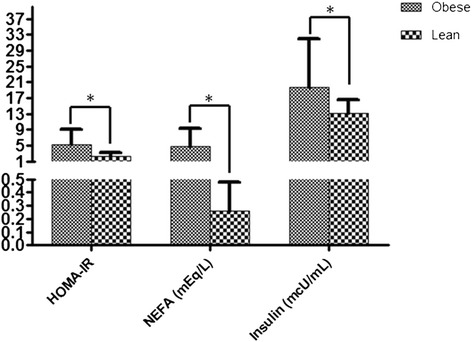
**Determination of metabolic markers.** The metabolic study reflected that in comparison to obese and lean individuals had significantly higher serum levels (*p <0,000) of HOMA-IR, basal NEFAs (mEq/L), basal glycemia (mg/dL) (see Table 1) and basal insulin (mcU/mL).

Insulin levels, HOMA-IR and NEFA measurements are the markers we used to determine the association between obesity and the role of the inflammatory process. The concentration levels of these three markers have been significantly greater (p <0,000) in the obese than in persons of normal weight. Insulin 19,7 +/−13,2 mcU/mL *vs* 12,1+/− 3,4 mcU/mL, HOMA-IR 5.3 +/−3.7 *vs* 2.4+/−0.7 and NEFA 4,7 +/−4,7 mEq/L *vs* 0.21+/− 0.25 mEq/L, respectively (Figure 3).

## Discussion

Obesity is the consequence of an augment in adipose tissue, with accompanying alteration of the immune response, resulting in an increase in the number of macrophages in the AT, insulin resistance and hyperinsulinemia, elevated NEFA levels as well as elevated pro- inflammatory mediators (IL-1, IL-6 y TNF-α) [44-46].

Our study found: greater percentage of TLR4-CD14 expression and mean fluorescence index in monocytes, along with elevated levels of IL-6 and TNF α in obese adults compared to normal weight subjects. This data coincides with the work done by Ahmad *et al*. [47].

The patients in this study, although non-diabetic obese, presented insulin resistance, according to HOMA. The elevation of this hormone is an attempt to maintain the concentration of glucose within normal levels. But insulin resistance elevates NEFAs [48]. The obese patients in our study show NEFA levels 18 times higher than the normal weight individuals. NEFAs are TLR4 agonists that might be triggering a low-grade inflammatory response, as evidenced by the seric concentrations of TNF-α and IL-6 [23]. Delving deeper into the identification of the types of fatty acids that are being released will permit greater understanding of the pro-inflammatory/metabolic feedback that we found in these patients. Adipose tissue maintains a proportion between saturated fatty acids, palmitic and stearic acids at 22%, and monounsaturated oleic at 50%. This proportion is maintained in the adipose tissue of both obese and normal weight individuals [49]. It has been shown that long chain saturated fatty acids are the ones that produce the inflammatory response and they have the capacity to express TLR-4 and TLR-2 in monocytes *in vitro* which would explain, in part, the increased expression in the obese group.

Although the obese group were not diabetic, it would be interesting to do research *in vitro* with TLR-4 and TLR-2 induction since high levels of glucose of 270 mg/dl, along with high NEFA concentrations, augment the induction effect of TLR-4, the release of Reactive Oxygen Species (ROS), and inflammatory cytokines in human monocytes *in vitro* [50]. On the other hand, it has been demonstrated that obese individuals maintain a state of constant stress mediated by the excess release of pro-inflammatory cytokines such as ROS, in addition to physiological stress, in which the neurotransmitter noradrenalin has a direct effect on the immune system via specific receptors that set up another vicious feedback cycle between the cells of the immune system, the endocrine and the central nervous system [51].

Conclusion: Adipose tissue used to be thought of as mere storage of fats and energy, but it has been revealed to be an important neuro-immune-endocrine organ. Immune cells, stimulated by NEFAs, produce pro-inflammatory cytokines, which have a direct effect on oxidating radicals that directly target the release of noradrenalin. This in turn, reactivates the vicious cycle of low-grade chronic inflammation, as is now described in obesity.
